# Dataset of non-timber forest products use and impacts of recent climate change in the Upper Madi Watershed, Nepal

**DOI:** 10.1016/j.dib.2020.106404

**Published:** 2020-10-10

**Authors:** Lila Jung Gurung, Kelly K. Miller, Susanna Venn, Brett A. Bryan

**Affiliations:** Centre for Integrative Ecology, School of Life and Environmental Sciences, Deakin University, Burwood, Victoria, Australia

**Keywords:** Non-timber forest products, Ecosystem services, Climate change, Mountains, People's perception

## Abstract

This dataset presents data collected from household surveys from Upper Madi Watershed of Nepal describing the benefits of non-timber forest products (NTFPs) to people of mountain ecosystems, their perceptions of climate change, and perceived impacts of climate change on NTFPs ecosystem services. The data were collected from 278 households that were randomly selected from the four villages in the watershed during the period September to December 2019. The survey assessed socio-demographic information; collected and utilized NTFPs; perceptions of climate change, and; perceived impacts of climate change on NTFPs ecosystem services. These data are important in understanding the benefits of non-timber forest products in mountain ecosystems and the impacts of climate change as the benefits and impacts are currently not well understood. The data will be helpful in formulation and implementation of adaptation strategies to sustain the supply, protection, and management of NTFPs in mountain ecosystems.

## Specifications Table

**Table:** 

Subject	Ecosystem services
Specific subject area	Non-timber forest products, Climate Change, Ecosystem Services, Mountain Ecosystems
Type of data	Primary data, Table
How data were acquired	Data were collected using a structured face to face household survey. The questionnaire is provided as a supplementary file
Data format	Raw Analysed
Parameters for data collection	The data were collected from the Upper Madi Watershed of Nepal. 278 households were surveyed.
Description of data collection	Data were collected from household surveys using structured questionnaires in Upper Madi Watershed of Nepal. Participants were selected using random sampling.
Data source location	Institution: Deakin University City/Town/Region: Melbourne Country: Australia
Data accessibility	With the article

## Value of the Data

• These data are important for understanding the benefits of non-timber forest products to mountain communities and the impacts of recent climate change as the benefits and impacts of climate change are not well understood.
• The data can benefit different stakeholders such as policymakers, practitioners in formulation and implementation of adaptation strategies to sustain supply, protection, and management of NTFPs in mountain ecosystems.
• Researchers in the field of climate change impacts, non-timber forest products and mountain ecosystems can use these data to compare with similar studies in the mountains elsewhere or supporting systematic reviews in the future.

## 1 Data Description

The dataset provides information on data collected from 278 household surveys on the benefits of non-timber forest products, perceptions of climate change within mountain communities and impacts of climate change on non-timber forest product ecosystem services. The survey data include the following sections: a) socio-demographic information of respondents including age, gender, ethnicity, educational background and occupation b) utilization of non-timber forest products by mountain communities c) perceptions of climate change d) Perceived impacts of climate change on non-timber forest products ecosystem services. The questionnaire is provided as a supplementary file. Social-demographic characteristics are presented in Table 1.

**Table 1:** Socio-demographic characteristics of respondents (*n* = 278).

Characteristics	Category	Frequency	Proportion (%)
Gender	Male	174	62.6
	Female	104	37.4
Age (Years)	18–35	16	5.8
	36–60	122	43.9
	>60	140	50.3
Ethnicity	Gurung	219	78.8
	Dalit	54	19.4
	Others	5	1.8
Respondents' education	No formal education	171	61.5
	Primary	97	34.9
	Secondary	10	3.6
	Tertiary	0	0.0
Respondents' occupation	Farmer	271	97.5
	Business	7	2.5
Annual household income (Nepalese Rupee-NPR)	No income	1	0.3
	<25,000	24	8.6
	25,000-50,000	75	27.0
	51,000-100,000	83	29.9
	>100,000	95	34.2

The details of non-timber forest product use in the Upper Madi Watershed of Nepal, number of non-timber forest plant species, purpose of NTFPs collection, respondent perceptions of climate change, perceived impacts of climate change on non-timber forest products ecosystem services are described in Table 2, Table 3, Table 4, Table 5, Table 6, Table 7, Table 8. Data are provided as a supplementary file.

**Table 2:** Access of respondents to forest (*n* = 278).

	Responses	Proportion (%)
Does your family have access to forest?	Yes	100
	No	0

**Table 3:** Respondents reporting the use of non-timber forest products among the mountain communities.

	Responses	
Types of NTFPs collection	Frequency	Proportion (%)
Fodder	211	76
Fuelwood	278	100
Medicinal Plants	101	36
Bamboo products	210	75
Nettle products	167	60
Wild Fruit	228	82
Wild Vegetable	237	85
Ornamental Plants	206	74
Agricultural Tools	114	41
Ritual Plants	32	12

**Table 4:** Name and number of plant species used in each NTFPs type.

Sp. No	Local/Common Name	English Name	Scientific Name
Fodder			
1	Badahar	Monkey Jack	*Artocarpus lakoocha*
2	Chhomu/Ghude	Himalayan Small Bamboo	*Thamnocalamus aristatus*
3	Kalahkulu/Tusaro	Not available	*Not available*
4	Kamu/Tite	Himalayan Small Bamboo	*Drepanostachyum falcatum*
5	Khorsan Chhi	Not available	*Not available*
6	Malkaji/Gophla	Not available	*Holboellia latifolia*
7	Miphu Chhi/Liso	Not available	*Scurrula parasitica*
8	Ng-ra/ Dudhilo	Not available	*Ficus neriifolia*
9	Ngsi/Phalat	Blue Japanese Oak	*Quercus glauca*
10	Nishi/Kharsu	Brown Oak of Himalaya	*Quercus semicarpifolia*
11	Nuri chhi	Not available	*Not available*
12	Odu Chhi/Gogan	Not available	*Saurauia napaulensis*
13	Pleta Chhi/Chiple	Not available	*Pouzolzia sanguinea*
14	Plowu/Malinge	Himalayan Small Bamboo	*Himalayacalamus cupreus*
15	Prapri/Firfire Ghans	Not available	*Hydrangea robusta*
16	Pudki/Pachpate	Not available	*Sausurea pennata*
17	Purichhi/Chuletro	Not available	*Brassaiopsis hainla*
18	RuRu	Not available	*Not available*
19	Tuhuru/Guyeli	Oleaster	*Elaeagnus latifolia*
20	Tumu/Thotne	Not available	*Polygonum molle*
Fuelwood			
1	Changi/Loth Salla	Himalayan Yew	*Taxus wallichiana*
2	Chhodi/Bilaune	Not available	*Maesa chisia*
3	Chhomu/Ghude	Himalayan Small Bamboo	*Thamnocalamus aristatus*
4	Chohsi/Jhyano	Not available	*Eurya acuminata*
5	Chyarbu/Paiyu	Himalayan Cherry	*Prunus cerasoides*
6	Chyarsi/Angeri	Lyonia	*Lyonia ovalifolia*
7	Ghyosi/Uttis	Alder	*Alnus nepalensis*
8	Herah/Malah	Not available	*Viburnum mullaha*
9	Newa Si/Ashare	Not available	*Viburnum nervosum*
10	Ngsi/Phalat	Blue Japanese Oak	*Quercus glauca*
11	Palah/Aiselu	Golden Evergreen Raspberry	*Rubus ellipticus*
12	Phusre	Not available	*Hydrangea heteromalla*
13	Plowu/Malinge	Himalayan Small Bamboo	*Himalayacalamus cupreus*
14	Poritah/Laligurans	Rhododendron	*Rhododendron arboreum*
15	Rakchan	Not available	*Daphniphyllum himalense*
16	Syona/Champ	Magnolia	*Magnolia champaca*
17	Tisya/Bhutro	Nepal Barberry	*Berberis aristata*
18	Tiuru/Bhakimlo	Chinese Sumac	*Rhus javanica*
19	Tohsi/ Kali Kath	Not available	*Myrsine semiserrata*
20	Tuhuru/Guyeli	Oleaster	*Elaeagnus latifolia*
Medicinal			
1	Banmara	Crofton Weed	*Ageratina adenophora*
2	Changi/Loth Salla	Himalayan Yew	*Taxus wallichiana*
3	Cheuri/Titepati	Mug Wart	*Artemisia dubia*
4	Chhyodomai /Bojho	Sweet Flag Calamus Root	*Acorus calamus*
5	Ghodtapre	Water Pennywort	*Centella asiatica*
6	Ghurbasan/Pakhanbed	Hairy Bergenia	*Bergenia ciliata*
7	Gurja	Heart-leaved Moonseed	*Tinospora cordifolia*
8	Hardjorne	Orchid	*Dendrobium amoenum*
9	Hey Nhu/ Ban Lasun	Wild Garlic	*Allium wallichii*
10	Heytanda/Lekbadmali	Not available	*Polygonum*
11	Kafal	Box Myrtle Bayberry	*Myrica esculenta*
12	Kudki	Not available	*Neopicrorhiza scrophulariiflora*
13	Kudu/Siltimur	Pepper	*Lindera neesiana*
14	Megai/Bikh	Not available	*Aconitum ferox*
15	Nihi Polhu/Allo	Himalayan Nettle	*Girardinia diversifolia*
16	Nirmashi	Not available	*Aconitum gammiei*
17	Okhar	Walnut	*Juglans regia*
18	Olmi/Halhale	Yellow Doek	*Rumex nepalensis*
19	Palah/Aiselu	Golden Evergreen Raspberry	*Rubus ellipticus*
20	Panch Amle	Orchid	*Dactylorhiza hatagirea*
21	Pipla	Wild Pepper	*Piper mullesua*
22	Poritah/Laligurans	Rhododendron	*Rhododendron arboreum*
23	Pruma/Timur	Nepal Pepper	*Zanthoxylum armatum*
24	Pudunchale/Padamchal	Himalayan Rhubarb	*Rheum australe*
25	Satwa/Satuwa	Not available	*Paris polyphylla*
26	Teedo/Chiraito	Chiretta	*Swertia chirayita*
27	Thakailo	Not available	*Cirsium verutum*
28	Tisya/Bhutro	Nepal Barberry	*Berberis aristata*
29	Tiuru/Bhakimlo	Chinese Sumac	*Rhus javanica*
30	Yarshya Gumba	Caterpillar Fungus	*Cordypses sinensis*
Nettle			
1	Nihi Polhu/Allo	Himalayan Nettle	*Girardinia diversifolia*
Bamboo			
1	Chhomu/Ghude	Himalayan Small Bamboo	*Thamnocalamus aristatus*
2	Chigar	Himalayan Small Bamboo	*Borinda chigar*
3	Kamu/Tite	Himalayan Small Bamboo	*Drepanostachyum falcatum*
4	Misur	Himalayan Small Bamboo	*Not available*
5	Pirma	Himalayan Small Bamboo	*Not available*
6	Plowu/Malinge	Himalayan Small Bamboo	*Himalayacalamus cupreus*
7	Syul	Himalayan Small Bamboo	*Not available*
Wild fruit			
1	Chhyaudi	Not available	*Not available*
2	Chutro	Common Barberry	*Berberis asiatica*
3	Chyarbu/Paiyu	Himalayan Cherry	*Prunus cerasoides*
4	Herah/Malah	Not available	*Viburnum mullaha*
5	Kabu	Not available	*Not available*
6	Kafal	Box Myrtle Bayberry	*Myrica esculenta*
7	Kasi/Katus	Nepal Chestnut	*Castanopsis indica*
8	Malkaji/Gophla	Not available	*Holboellia latifolia*
9	Meh Palah/Kimbu	Black Mulberry	*Morus nigra*
10	Miphu Chhi/Liso	Not available	*Scurrula parasitica*
11	Mowa/Khanayo	Nepal Fodder Fig	*Ficus semicordata*
12	Naljyo	Not available	*Not available*
13	Noulukujyu/Khiramlo	Not available	*Polygonatum verticillatum*
14	Okhar	Walnut	*Juglans regia*
15	Palah/Aiselu	Golden Evergreen Raspberry	*Rubus ellipticus*
16	Puri	Himalayan Bird Cherry	*Prunus napaulensis*
17	Sisi	Not available	*Not available*
18	Tehwu	Not available	*Not available*
19	Tisya/Bhutro	Nepal Barberry	*Berberis aristata*
20	Toju/Golkakri	Creeping Cucumber	*Solena heterophylla*
21	Tuhuru/Guyeli	Oleaster	*Elaeagnus latifolia*
Wild vegetables			
1	Ban Temi/Ban Tarul	Potato Yam	*Dioscorea bulbifera*
2	Chhomu/Ghude	Himalayan Small Bamboo	*Thamnocalamus aristatus*
3	Hey Nhu/ Ban Lasun	Wild Garlic	*Allium wallichii*
4	Jali Chyau	Mushroom	*Morchella conica*
5	Jibre Saag	Adder's Tongue	*Ophioglossum vulgatum*
6	Kalunge Chyau	Mushroom	*Termitomyces eurhizus*
7	Kanya Chyau	Mushroom	*Pleurotus nepalensis*
8	Laule Tah/Dhaga Saag	Not available	*Rheum*
9	Lotah/Niuro	Edible Fern Shoot	*Dryopteris cochleata*
10	Maye Tah/Makai Saag	Not available	*Not available*
11	Mirge Chyau	Mushroom	*Lentinula edodes*
12	Nagroom Chyau	Mushroom	*Grifola frondosa*
13	Olmi/Halhale	Yellow Doek	*Rumex nepalensis*
14	Pahi Tah	Not available	*Not available*
15	Plema/Chiple Chyau	Mushroom	*Suillus granulatus*
16	Plowu/Malinge	Himalayan Small Bamboo	*Himalayacalamus cupreus*
17	Puchutohru/ Kurilo	Wild Asparagus	*Asparagus racemosus*
18	Rato Chayu	Mushroom	*Laetiporus sulphureus*
19	Thankre Chyau	Mushroom	*Clavaria cristata*
Ornamental			
1	Ghurbasan/Pakhanbed	Hairy Bergenia	*Bergenia ciliata*
2	Mahu Tah	Himalayan Primrose	*Primula denticulata*
3	Poritah/Laligurans	Rhododendron	*Rhododendron arboreum*
4	Sal Tah/Sungava	Orchid	*Coelogyne cristata*
5	Syona/Champ	Magnolia	*Magnolia champaca*
Agricultural tools			
1	Changi/Loth Salla	Himalayan Yew	*Taxus wallichiana*
2	Chyarbu/Paiyu	Himalayan Cherry	*Prunus cerasoides*
3	Kyosi/Chilaune	Needle Wood	*Schima wallichii*
4	Newa Si/Ashare	Not available	*Viburnum nervosum*
5	Ngsi/Phalat	Blue Japanese Oak	*Quercus glauca*
6	Poritah/Laligurans	Rhododendron	*Rhododendron arboreum*
Ritual			
1	Ban Temi/Ban Tarul	Potato Yam	*Dioscorea bulbifera*
2	Cheuri/Titepati	Mug Wart	*Artemisia dubia*
3	Chhomu/Ghude	Himalayan Small Bamboo	*Thamnocalamus aristatus*
4	Chhyou Mhai/Nagbeli	Clubmoss	*Lycopodium clavatum*
5	Chhyu Tah	Not available	*Not available*
6	Chyarbu/Paiyu	Himalayan Cherry	*Prunus cerasoides*
7	Herah/Malah	Not available	*Viburnum mullaha*
8	Kamu/Tite	Himalayan Small Bamboo	*Drepanostachyum falcatum*
9	Khel/Bhojpatra	Himalayan Silver Birch	*Betula utilis*
10	Lerah	Not available	*Not available*
11	Neri/Kukurdaino	Green Briers	*Smilax aspera*
12	Ng-ra/ Dudhilo	Not available	*Ficus neriifolia*
13	Plowu/Malinge	Himalayan Small Bamboo	*Himalayacalamus cupreus*
14	Pruma/Timur	Nepal Pepper	*Zanthoxylum armatum*
15	Puchutohru/ Kurilo	Wild Asparagus	*Asparagus racemosus*
16	RuRu	Not available	*Not available*
17	Siuru/Dhupi	Himalayan Pencil Cedar	*Juniperus communis*
18	Thundura Tah/Khaldi	Not available	*Bistorta*
19	Tili Tah	Not available	*Not available*
20	Tiuru/Bhakimlo	Chinese Sumac	*Rhus javanica*
21	Tu Nho/Dubo	Bermuda Grass	*Cynodon dactylon*

**Table 5:** Purpose of NTFPs collection (*n* = 278).

	Responses	Proportion (%)
Purpose of NTFPs collection	Household use only	42.1
	Both for sale and household use	57.9

**Table 6:** Extent to which respondents agree that climate change is occurring (*n* = 278).

Agreement that climate change is occurring	Responses	Proportion (%)
Extent of agreement on the statement “Climate change is happening in your area”	Strongly disagree	0
	Disagree	0
	Not sure	13
	Agree	86
	Strongly agree	1

**Table 7:** Perception of respondents (*n* = 278) on various indicators of climate change.

	Responses				
Perception of change in climate indicators	Significantly Decreased (%)	Decreased (%)	No Change (%)	Increased (%)	Significantly Increased (%)
Maximum summer temperature	0	0	14	86	0
Maximum winter temperature	0	16	33	51	0
Minimum summer temperature	0	0	23	77	0
Minimum winter temperature	0	35	27	38	0
Summer rainfall amount	0	7.9	24.1	67.6	0.4
Winter rainfall amount	0	75.9	19.8	4.3	0
Summer drought	0	7.2	69.1	23.7	0
Winter drought	0	3.2	22.7	74.1	0
Snow fall	0.3	95	4	0.7	0
Hailstorms	0	19.8	21.6	58.3	0.3
Strong wind	0.4	18	40.6	41	0
Landslides	0	17.6	35.6	46.5	0.3
Floods	0	17.3	36	46.4	0.3
Forest fire	0	21	77	2	0
Pest and insects	0	0.4	2.5	96.4	0.7
Invasive plant species	0	0	0.4	91	8.6

**Table 8:** Perceived impacts of climate change and extreme events on NTFPs.

	Responses				
	Significantly Decreased (%)	Decreased (%)	No change (%)	Increased (%)	Significantly increased (%)
Impacts on fodder					
Forest fire	0	0	98	2	0
Drought	0	0	93	7	0
Change in precipitation	0	0	92	8	0
Change in temp. pattern	0	0	92	8	0
Floods	0	0	90	10	0
Landslides	0	0	81	19	0
Wind	0	0	52	48	0
Pest and insects	0	0	41	59	0
Hailstorm	0	0	31	69	0
Invasive plant sp.	0	0	20	78	2
Impacts on fuelwood					
Forest fire	0	0	98	2	0
Drought	0	0	97	3	0
Change in precipitation	0	0	95	5	0
Change in temp. pattern	0	0	94	6	0
Floods	0	0	91	9	0
Landslides	0	0	83	17	0
Hailstorm	0	0	61	39	0
Wind	0	0	59	41	0
Invasive plant sp.	0	0	26	74	0
Pest and insects	0	0	7	93	0
Impacts on medicinal plants					
Forest fire	0	0	100	0	0
Landslides	0	0	99	1	0
Drought	0	0	95	5	0
Invasive plant sp.	0	0	93	7	0
Floods	0	0	91	9	0
Change in precipitation	0	0	78	22	0
Change in temp. pattern	0	0	74	26	0
Wind	0	0	71	29	0
Pest and insects	0	0	65	35	0
Hailstorm	0	0	49	51	0
Impacts on nettle products					
Forest fire	0	0	100	0	0
Drought	0	0	99	1	0
Floods	0	0	87	13	0
Landslides	0	0	78	22	0
Change in precipitation	0	0	77	23	0
Change in temp. pattern	0	0	76	24	0
Wind	0	0	65	35	0
Pest and insects	0	0	53	47	0
Hailstorm	0	0	50	50	0
Invasive plant sp.	0	0	28	72	0
Impacts on bamboo products					
Forest fire	0	0	100	0	0
Floods	0	0	99	1	0
Drought	0	0	96	4	0
Landslides	0	0	94	6	0
Change in precipitation	0	0	77	23	0
Invasive plant sp.	0	0	70	30	0
Change in temp. pattern	0	0	69	31	0
Hailstorm	0	0	63	37	0
Wind	0	0	57	43	0
Pest and insects	0	0	41	59	0
Impacts on agricultural tools					
Change in temp. pattern	0	0	100	0	0
Drought	0	0	100	0	0
Forest fire	0	0	100	0	0
Change in precipitation	0	0	99	1	0
Floods	0	0	98	2	0
Landslides	0	0	98	2	0
Invasive plant sp.	0	0	86	14	0
Hailstorm	0	0	83	17	0
Pest and insects	0	0	79	21	0
Wind	0	0	63	37	0
Impacts on wild fruit					
Forest fire	0	0	100	0	0
Floods	0	0	99	1	0
Drought	0	0	98	2	0
Change in precipitation	0	0	97	3	0
Landslides	0	0	96	4	0
Change in temp. pattern	0	0	96	4	0
Invasive plant sp.	0	0	82	18	0
Wind	0	0	59	41	0
Pest and insects	0	0	50	50	0
Hailstorm	0	0	42	58	0
Impacts on wild vegetables					
Forest fire	0	0	100	0	0
Drought	0	0	98	2	0
Change in temp. pattern	0	0	95	5	0
Change in precipitation	0	0	95	5	0
Floods	0	0	93	7	0
Landslides	0	0	86	14	0
Wind	0	0	63	37	0
Pest and insects	0	0	57	43	0
Hailstorm	0	0	42	58	0
Invasive plant sp.	0	0	12	88	0
Impacts on ornamental plants					
Forest fire	0	0	100	0	0
Drought	0	0	99	1	0
Floods	0	0	99	1	0
Change in temp. pattern	0	0	98	2	0
Change in precipitation	0	0	97	3	0
Landslides	0	0	97	3	0
Invasive plant sp.	0	0	92	8	0
Wind	0	0	59	41	0
Pest and insects	0	0	48	52	0
Hailstorm	0	0	44	56	0
Impacts on ritual plants					
Forest fire	0	0	100	0	0
Floods	0	0	97	3	0
Drought	0	0	97	3	0
Landslides	0	0	94	6	0
Change in temp. pattern	0	0	90	10	0
Change in precipitation	0	0	90	10	0
Pest and insects	0	0	77	23	0
Invasive plant sp.	0	0	77	23	0
Wind	0	0	52	48	0
Hailstorm	0	0	42	58	0

## 2 Survey Design, Materials and Methods

This research was based on primary data collection through a household survey [1], [2], [3], [4] during the period September to December 2019. In our survey the unit of analysis was the household and the household head or his/her representative was the respondent. A complete list of 909 households from the four villages (Sikles, Parche, Khilang and Tangting) in the Upper Madi watershed was first collected from the election commission of Nepal. Sample size of 278 households was calculated out of 909 households by using the formula proposed by [5] to provide a statistically significant sample from the target population of the study area [6,7]. Our sample represents approximately 31% of the total households in the study area. The Statistical Package for the Social Sciences (SPSS) was used to randomly select 278 households from the household list. We had contacted 278 householders and all of them had responded, most of the respondents were household heads and some were representatives of the households where the heads were absent. Face to face interviews were conducted with household heads (or their representatives) using a structured questionnaire containing both closed and open-ended questions [1]. To check for clarity and understanding of the survey questions, 10 households were pre-tested [1,8] prior to survey implementation. 5-point Likert scales [1] were used to quantify the local perceptions of climate change and perceived impacts on NTFP ecosystem services. The quantitative data obtained from household surveys were analyzed using SPSS and Microsoft Excel to obtain descriptive statistics such as frequencies and percentages of responses. The Dictionary of Nepalese Plant Names [9] was used to identify the botanical and common English names of most plant species as local common names collected from household survey were in the Gurung language.

## Ethics Statement

Ethical approval was obtained from the faculty of Science Engineering and Built Environment Human Ethics Advisory Group, Deakin University (reference number STEC-31–2019-GURUNG). Respondents’ participation was completely agreed, voluntary, and anonymous.

## Authors’ contribution

Lila Jung Gurung: Conceptualization, methodology, data collection, and writing of the manuscript.

Kelly Miller, Susanna Venn, and Brett A Bryan: Conceptualization, methodology, review and editing the manuscript.

## Declaration of Competing Interest

The authors declare that they have no known competing financial interests or personal relationships which have, or could be perceived to have, influenced the work reported in this article.

## Appendix Supplementary materials

Image, application 1

Image, application 2
