# Supplementary material for: Dataset of non-timber forest products use and impacts of recent climate change in the Upper Madi Watershed, Nepal
Source: Data Brief. 2020 Oct 10;33:106404. doi: 10.1016/j.dib.2020.106404 (PMC7569287; doi:10.1016/j.dib.2020.106404)
Supplement: Supplementary file 2 [file mmc2.docx]

**Questionnaires**

**A: Socio Demographic Information**

Please tick the relevant boxes.

1. What is your gender? Male 🞏 Female 🞏 Other 🞏
2. Which age group do you belong to?

Age: 18-35 years 🞏 36- 60 years 🞏 Above 60 years 🞏

1. Which ethnic/caste group do you belong to? Gurung 🞏 Dalit 🞏 Others 🞏
2. What is your level of education? No formal education 🞏 Primary Education 🞏 Secondary Education 🞏 Tertiary Education 🞏
3. What is your occupation? Employed 🞏 Farmer 🞏 Business 🞏 Others 🞏
4. What is your annual household income (In NPR)? No monetary income 🞏 below 25,000 🞏 25,000-50,000 🞏 51,000-100,000 🞏 above 100,000 🞏

**B: NTFPs and Livelihood Information**

1. Does your family have access to forest? Yes 🞏 No 🞏
2. What types of NTFPs do you collect? Tick all that apply.

Fodder 🞏 Fuelwood 🞏 Medicinal Plants 🞏 Nettle plant 🞏 Bamboo (*Nigalo*) 🞏 Wild Fruit 🞏 Wild Vegetables 🞏 Ornamental Plants 🞏 Agricultural Tools 🞏 Ritual Plants 🞏 Others 🞏

1. If you collect fodder, what species are they?
2. If you collect fuelwood, what species are they?
3. If you collect medicinal plants, what species are they?
4. If you collect nettle, what species are they?
5. If you collect bamboo (*Nigalo*), what species are they?
6. If you collect wild fruits, what species are they?
7. If you collect wild vegetables, what species are they?
8. If you collect ornamental plants, what species are they?
9. If you collect materials to make agricultural tools, what species are they?
10. If you collect ritual plants, what species are they?
11. Why do you collect the NTFPs? For household use 🞏 To sell 🞏 for both 🞏

**C: Perception of Climate Change**

1. To what extent do you agree with the statement “Climate Change is happening in your area”? Please tick the response below.
2. Strongly Disagree 🞏
3. Disagree 🞏
4. Not sure 🞏
5. Agree 🞏
6. Strongly Agree 🞏
7. How do you compare the recent (past five years) temperature to the temperature in the past 20-30 years? Please tick the relevant box below. (1-Significantly Decreased, 2-Decreased, 3-No Change, 4-Increased, 5-Significantly Increased)

|  | **Summer** | **Winter** |
| --- | --- | --- |
| Maximum temperature | 1🞏 2🞏 3🞏 4🞏 5🞏 | 1🞏 2🞏 3🞏 4🞏 5🞏 |
| Minimum temperature | 1🞏 2🞏 3🞏 4🞏 5🞏 | 1🞏 2🞏 3🞏 4🞏 5🞏 |

1. How do you compare the recent (past five years) rainfall to the rainfall in the past 20-30 years? Please tick the response below. (1-Significantly Decreased, 2-Decreased, 3-No Change, 4-Increased, 5-Significantly Increased)

|  | **Summer** | **Winter** |
| --- | --- | --- |
| Rainfall amount | 1🞏 2🞏 3🞏 4🞏 5🞏 | 1🞏 2🞏 3🞏 4🞏 5🞏 |

1. How do you compare your experience of recent (past five years) drought to the drought in the past 20-30 years? Please tick the response below. (1-Significantly Decreased, 2-Decreased, 3-No Change, 4-Increased, 5-Significantly Increased)

|  | **Summer** | **Winter** |
| --- | --- | --- |
| Drought events | 1🞏 2🞏 3🞏 4🞏 5🞏 | 1🞏 2🞏 3🞏 4🞏 5🞏 |

1. What do you think about the recent (last five years) frequency of snowfall, hailstorm and strong wind in comparison to past 20-30 years? Please tick the response below. (1-Significantly Decreased, 2-Decreased, 3-No Change, 4- Increased, 5-Significantly Increased)

| Snowfall | 1🞏 2🞏 3🞏 4🞏 5🞏 |
| --- | --- |
| Hailstorm | 1🞏 2🞏 3🞏 4🞏 5🞏 |
| Strong wind | 1🞏 2🞏 3🞏 4🞏 5🞏 |

1. What do you think about the recent (last five years) hazards in comparison to past 20-30 years? Please tick the response below. (1-Significantly Decreased, 2-Decreased, 3-No Change, 4- Increased, 5-Significantly Increased)

| Landslides | 1🞏 2🞏 3🞏 4🞏 5🞏 |
| --- | --- |
| Floods | 1🞏 2🞏 3🞏 4🞏 5🞏 |
| Forest fires | 1🞏 2🞏 3🞏 4🞏 5🞏 |
| Pests and insects | 1🞏 2🞏 3🞏 4🞏 5🞏 |
| Invasive plant species | 1🞏 2🞏 3🞏 4🞏 5🞏 |

**D. Knowledge of Climate Change Impacts on NTFPs**

1. How do you perceive the impacts of climate change on availability of following NTFPs over the last 20-30 years? Please tick the response below. (0: Not applicable 1: Significantly decreased, 2: Decreased, 3: No change, 4: Increased, 5 Significantly increased)

|  | 26.1 Fodder | 26.2 Fuelwood | 26.3 Medicinal Plants |
| --- | --- | --- | --- |
| Change in overall temperature patterns | 0🞏1🞏2🞏3🞏4🞏5🞏 | 0🞏1🞏2🞏3🞏4🞏5🞏 | 0🞏1🞏2🞏3🞏4🞏5🞏 |
| Change in overall precipitation patterns | 0🞏1🞏2🞏3🞏4🞏5🞏 | 0🞏1🞏2🞏3🞏4🞏5🞏 | 0🞏1🞏2🞏3🞏4🞏5🞏 |
| Droughts | 0🞏1🞏2🞏3🞏4🞏5🞏 | 0🞏1🞏2🞏3🞏4🞏5🞏 | 0🞏1🞏2🞏3🞏4🞏5🞏 |
| Landslides | 0🞏1🞏2🞏3🞏4🞏5🞏 | 0🞏1🞏2🞏3🞏4🞏5🞏 | 0🞏1🞏2🞏3🞏4🞏5🞏 |
| Forest fire | 0🞏1🞏2🞏3🞏4🞏5🞏 | 0🞏1🞏2🞏3🞏4🞏5🞏 | 0🞏1🞏2🞏3🞏4🞏5🞏 |
| Strong wind | 0🞏1🞏2🞏3🞏4🞏5🞏 | 0🞏1🞏2🞏3🞏4🞏5🞏 | 0🞏1🞏2🞏3🞏4🞏5🞏 |
| Floods | 0🞏1🞏2🞏3🞏4🞏5🞏 | 0🞏1🞏2🞏3🞏4🞏5🞏 | 0🞏1🞏2🞏3🞏4🞏5🞏 |
| Hailstorm | 0🞏1🞏2🞏3🞏4🞏5🞏 | 0🞏1🞏2🞏3🞏4🞏5🞏 | 0🞏1🞏2🞏3🞏4🞏5🞏 |
| Pest and insects | 0🞏1🞏2🞏3🞏4🞏5🞏 | 0🞏1🞏2🞏3🞏4🞏5🞏 | 0🞏1🞏2🞏3🞏4🞏5🞏 |
| Invasive plant species | 0🞏1🞏2🞏3🞏4🞏5🞏 | 0🞏1🞏2🞏3🞏4🞏5🞏 | 0🞏1🞏2🞏3🞏4🞏5🞏 |

1. How do you perceive the impacts of climate change on availability of following NTFPs over last 20-30 years? Please tick the response below. (0: Not applicable 1: Significantly decreased, 2: Decreased, 3: No change, 4: Increased, 5 Significantly increased)

|  | 27.1 Nettle products | 27.2 Bamboo products | 27.3 Agricultural tools |
| --- | --- | --- | --- |
| Change in overall temperature patterns | 0🞏1🞏2🞏3🞏4🞏5🞏 | 0🞏1🞏2🞏3🞏4🞏5🞏 | 0🞏1🞏2🞏3🞏4🞏5🞏 |
| Change in overall precipitation patterns | 0🞏1🞏2🞏3🞏4🞏5🞏 | 0🞏1🞏2🞏3🞏4🞏5🞏 | 0🞏1🞏2🞏3🞏4🞏5🞏 |
| Droughts | 0🞏1🞏2🞏3🞏4🞏5🞏 | 0🞏1🞏2🞏3🞏4🞏5🞏 | 0🞏1🞏2🞏3🞏4🞏5🞏 |
| Landslides | 0🞏1🞏2🞏3🞏4🞏5🞏 | 0🞏1🞏2🞏3🞏4🞏5🞏 | 0🞏1🞏2🞏3🞏4🞏5🞏 |
| Forest fire | 0🞏1🞏2🞏3🞏4🞏5🞏 | 0🞏1🞏2🞏3🞏4🞏5🞏 | 0🞏1🞏2🞏3🞏4🞏5🞏 |
| Strong wind | 0🞏1🞏2🞏3🞏4🞏5🞏 | 0🞏1🞏2🞏3🞏4🞏5🞏 | 0🞏1🞏2🞏3🞏4🞏5🞏 |
| Floods | 0🞏1🞏2🞏3🞏4🞏5🞏 | 0🞏1🞏2🞏3🞏4🞏5🞏 | 0🞏1🞏2🞏3🞏4🞏5🞏 |
| Hailstorm | 0🞏1🞏2🞏3🞏4🞏5🞏 | 0🞏1🞏2🞏3🞏4🞏5🞏 | 0🞏1🞏2🞏3🞏4🞏5🞏 |
| Pest and insects | 0🞏1🞏2🞏3🞏4🞏5🞏 | 0🞏1🞏2🞏3🞏4🞏5🞏 | 0🞏1🞏2🞏3🞏4🞏5🞏 |
| Invasive plant species | 0🞏1🞏2🞏3🞏4🞏5🞏 | 0🞏1🞏2🞏3🞏4🞏5🞏 | 0🞏1🞏2🞏3🞏4🞏5🞏 |

1. How do you perceive the impacts of climate change on availability of followings NTFPs over last 20-30 years? Please tick the response below. (0: Not applicable 1: Significantly decreased, 2: Decreased, 3: No change, 4: Increased, 5 Significantly increased)

|  | 28.1 Wild fruits | 28.2 Wild vegetables | 28.3 Ornamental plants |
| --- | --- | --- | --- |
| Change in overall temperature patterns | 0🞏1🞏2🞏3🞏4🞏5🞏 | 0🞏1🞏2🞏3🞏4🞏5🞏 | 0🞏1🞏2🞏3🞏4🞏5🞏 |
| Change in overall precipitation patterns | 0🞏1🞏2🞏3🞏4🞏5🞏 | 0🞏1🞏2🞏3🞏4🞏5🞏 | 0🞏1🞏2🞏3🞏4🞏5🞏 |
| Droughts | 0🞏1🞏2🞏3🞏4🞏5🞏 | 0🞏1🞏2🞏3🞏4🞏5🞏 | 0🞏1🞏2🞏3🞏4🞏5🞏 |
| Landslides | 0🞏1🞏2🞏3🞏4🞏5🞏 | 0🞏1🞏2🞏3🞏4🞏5🞏 | 0🞏1🞏2🞏3🞏4🞏5🞏 |
| Forest fire | 0🞏1🞏2🞏3🞏4🞏5🞏 | 0🞏1🞏2🞏3🞏4🞏5🞏 | 0🞏1🞏2🞏3🞏4🞏5🞏 |
| Strong wind | 0🞏1🞏2🞏3🞏4🞏5🞏 | 0🞏1🞏2🞏3🞏4🞏5🞏 | 0🞏1🞏2🞏3🞏4🞏5🞏 |
| Floods | 0🞏1🞏2🞏3🞏4🞏5🞏 | 0🞏1🞏2🞏3🞏4🞏5🞏 | 0🞏1🞏2🞏3🞏4🞏5🞏 |
| Hailstorm | 0🞏1🞏2🞏3🞏4🞏5🞏 | 0🞏1🞏2🞏3🞏4🞏5🞏 | 0🞏1🞏2🞏3🞏4🞏5🞏 |
| Pest and insects | 0🞏1🞏2🞏3🞏4🞏5🞏 | 0🞏1🞏2🞏3🞏4🞏5🞏 | 0🞏1🞏2🞏3🞏4🞏5🞏 |
| Invasive plant species | 0🞏1🞏2🞏3🞏4🞏5🞏 | 0🞏1🞏2🞏3🞏4🞏5🞏 | 0🞏1🞏2🞏3🞏4🞏5🞏 |

1. How do you perceive the impacts of climate change on availability of following NTFPs over last 20-30 years? Please tick the response below. (0: Not applicable 1: Significantly decreased, 2: Decreased, 3: No change, 4: Increased, 5 Significantly increased)

|  | 29.1 Ritual Plants | 29.2 Others (………) |
| --- | --- | --- |
| Change in overall temperature patterns | 0🞏1🞏2🞏3🞏4🞏5🞏 | 0🞏1🞏2🞏3🞏4🞏5🞏 |
| Change in overall precipitation patterns | 0🞏1🞏2🞏3🞏4🞏5🞏 | 0🞏1🞏2🞏3🞏4🞏5🞏 |
| Droughts | 0🞏1🞏2🞏3🞏4🞏5🞏 | 0🞏1🞏2🞏3🞏4🞏5🞏 |
| Landslides | 0🞏1🞏2🞏3🞏4🞏5🞏 | 0🞏1🞏2🞏3🞏4🞏5🞏 |
| Forest fire | 0🞏1🞏2🞏3🞏4🞏5🞏 | 0🞏1🞏2🞏3🞏4🞏5🞏 |
| Strong wind | 0🞏1🞏2🞏3🞏4🞏5🞏 | 0🞏1🞏2🞏3🞏4🞏5🞏 |
| Floods | 0🞏1🞏2🞏3🞏4🞏5🞏 | 0🞏1🞏2🞏3🞏4🞏5🞏 |
| Hailstorm | 0🞏1🞏2🞏3🞏4🞏5🞏 | 0🞏1🞏2🞏3🞏4🞏5🞏 |
| Pest and insects | 0🞏1🞏2🞏3🞏4🞏5🞏 | 0🞏1🞏2🞏3🞏4🞏5🞏 |
| Invasive plant species | 0🞏1🞏2🞏3🞏4🞏5🞏 | 0🞏1🞏2🞏3🞏4🞏5🞏 |
